# American Institute of Homœopathy

**Published:** 1895-05

**Authors:** A. K. Crawford

**Affiliations:** Chairman; Chicago, Ill.


### AMERICAN INSTITUTE OF HOMŒOPATHY.

CHICAGO, ILL., May 8th, 1895.

EDITOR OF THE HOMŒOPATHIC PHYSICIAN :

The Transportation Committee begs to announce through your journal that special rates were conceded to members of the American Institute of Homœopathy for the annual meeting at Newport, R. I., June 20th to 29th, next, by the Boston Passenger Committee, and since concurred in by the Trunk Line Association and the Central Traffic Association. The rate authorized is one and one-third, certificate plan, for the round trip.

For New England the rates are as follows: Two cents per mile from points within twenty-five miles from Newport, with a mimimum rate of twenty-five cents. One dollar from points from twenty-five to thirty-three miles from Newport, and one and one-half cents per mile from points more than thirty-three miles from Newport.

In order to secure this reduction in fare, the members and delegates to the American Institute must bear in mind that it is on the certificate plan. This means that each person must purchase a first-class ticket to the place of meeting, for which he will pay the regular tariff fare, and upon request the ticket agent will issue to him a certificate of such purchase. When the member arrives in Newport he will hand his certificate to the Chairman of the Transportation Committee, who will attend to the red-tape requirements of the New England Committee. He will then be furnished with a return-trip ticket at one-third the amount paid going.

All the railroads running east from Chicago, St. Louis, and intermediate points are in the Associations which have granted these reduced fares, and as there is no "official route," each member has absolute freedom to choose his own line. Tickets from Chicago to Newport are $21 going and $7 returning by the differential roads, and by the other roads $23 going and $7.65 returning.

It is the intention at present for the Western delegates to leave Chicago and St. Louis on Monday, June 17th, to spend one day at some attractive resort *en route*, and arrive in New York on Wednesday, the 19th. They will proceed thence by the Fall River Line steamer, "The Plymouth," which leaves New York, Wednesday evening, at 6.30, from Pier 28, North River, Murray Street. This steamer has abundant accommodations for everybody, and state-rooms should be secured beforehand by applying to Mr. O. H. Taylor, agent of the Fall River Line, Pier 28, North River, New York City.

Members are requested to bear specially in mind the rigid rule of the Railroad Associations, that without a certificate no reduction in the return fare will be made.

Yours, fraternally,

A. K. Crawford, M. D.,

*Chairman.*

